# A Case of Amelanotic Plasmacytoid Melanoma with Strong CD138 Expression, Mimicking Multiple Myeloma – A Case Report 

**DOI:** 10.30699/ijp.2025.2040460.3354

**Published:** 2025-03-10

**Authors:** Ery Kus Dwianingsih, Yuni Artha Prabowo, Sofia Pranacipta, Shinta Andi Sarasati, Amri Wicaksono, Sekar Safitri, Eviana Norahmawati, Rahadyan Magetsari

**Affiliations:** 1 *Department of Anatomical Pathology, Faculty of Medicine, Public Health, and Nursing, Universitas Gadjah Mada, Yogyakarta, Indonesia*; 2 *Dr. Sardjito General Hospital, Jl Kesehatan No. 1, Sendowo, Yogyakarta, Indonesia*; 3 *Department of Surgery, Orthopaedic and Traumatology Division, Faculty of Medicine, Public Health and Nursing, Universitas Gadjah Mada, Yogyakarta, Indonesia*; 4 *Department of Radiology, Faculty of Medicine, Public Health and Nursing, Universitas Gadjah Mada, Yogyakarta, Indonesia *; 5 *Department of Anatomical Pathology, Faculty of Medicine, Universitas Brawijaya, Malang, Indonesia*

**Keywords:** Cutaneous Malignant, Hematologic Neoplasm, Syndecan-1

## Abstract

**Background & Objective:**

Melanomas have diverse pathological features that mimic other tumors, such as lymphoma, sarcoma, and even poorly differentiated carcinoma. The most recently identified variant, the plasmacytoid variant, is an uncommon variant that can appear as a solitary tumor or a metastatic disease. Due to its rarity, the epidemiologic profile of this variant is not well characterized. This case report illustrates a diagnostic challenge of plasmacytoid cell mimicker, which is rarely found in daily practice.

**Case Presentation:**

A 49-year-old man presented with multiple subcutaneous soft tissue nodules in the thoracic area and multiple pathological fractures in the left distal humerus and distal ulna. Clinical and radiological findings were suggestive of metastatic bone disease with differential diagnosis of multiple myeloma. Fine needle aspiration biopsy and histopathological findings were suggestive of multiple myeloma with differential diagnoses of metastatic carcinoma, rhabdomyosarcoma, and amelanotic melanoma. Thus, immunostaining for CD138, CK, desmin, vimentin, S-100, and HMB45 were requested and the results were compatible with the final diagnosis of amelanotic plasma melanoma.

**Conclusion:**

It is crucial to consider melanoma as one of the differential diagnoses of a tumor with plasmacytoid feature and CD138 positive staining as it can mimic multiple myeloma as demonstrated in this case report.

## Introduction

Melanomas have diverse pathological features that mimic many other tumors, such as lymphoma, sarcoma, and even poorly differentiated carcinoma. Malignant melanoma can present in various cutaneous and non-cutaneous sites, with numerous morphological variants, including spindle cell, epithelioid cell, clear cell, signet ring cell, small cell, chondroid, osteoid, myxoid, rhabdoid, and plasmacytoid melanoma. The most recently identified variant, the plasmacytoid variant, is an uncommon variant that can appear as a solitary tumor or a metastatic disease. The plasmacytoid variant, accompanied by amelanotic features and CD138 positivity, is a rare finding that is easily misdiagnosed as plasma cell neoplasm (1, 2). Due to its rarity, the epidemiologic profile of this variant is not well characterized. To our knowledge, only about 53 cases of plasmacytoid melanoma are described in the literature with cytological and biopsy findings of primary and metastatic lesions (1-5). This case report illustrates a diagnostic challenge of plasmacytoid cell mimicker, which is rarely found in daily practice. Written informed consent of the patient was obtained for disclosure of his medical conditions for study purposes.

## Case description

**Fig. 1:**
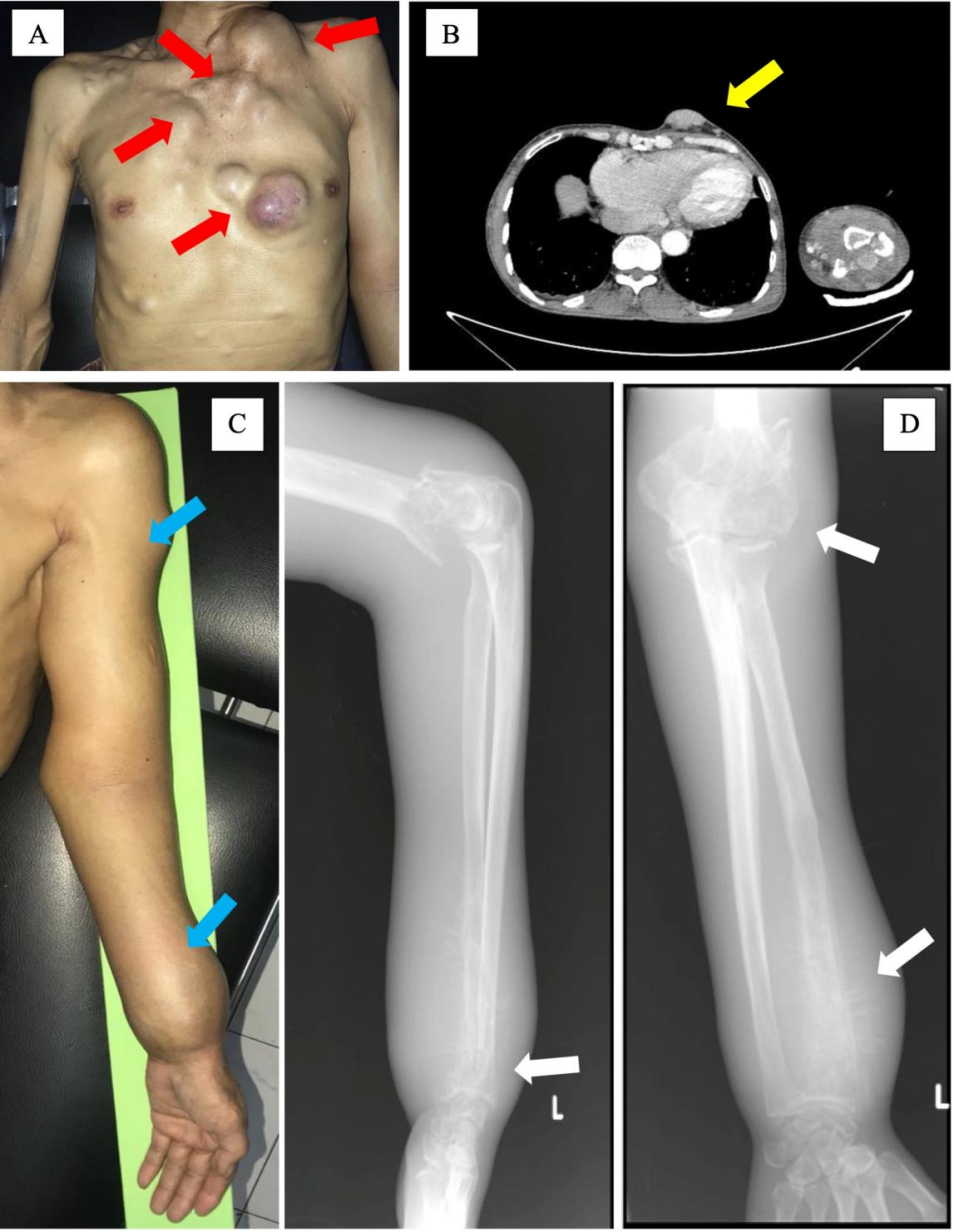
(A) Photograph of the patient showing multiple nodules in the chest (indicated by red arrows). (B) Multislice computed tomography (MSCT) image showing the subcutaneous soft tissue mass (indicated by the yellow arrow). (C) Photograph of the arm showing deformities caused by multiple fractures (blue arrows). (D) X-ray images confirm the fractures (white arrows).

A 49-year-old man presented with multiple subcutaneous soft tissue nodules in the thoracic area for one year (Figure 1A). The subcutaneous soft tissue nodules were confirmed with multislice computed tomography (MSCT), no other visceral involvement was observed (Figure 1B). He was admitted to the hospital due to multiple pathological fractures in the left distal humerus and distal ulna (Figure 1C). No skin or mucosal lesions were observed during the physical examination. X-ray examination showed multiple fractures in the left humerus and left ulna (Figure 1D). Clinical and radiological findings were suggestive of metastatic bone disease with differential diagnosis of multiple myeloma (MM) and metastatic bone disease. 

**Fig. 2:**
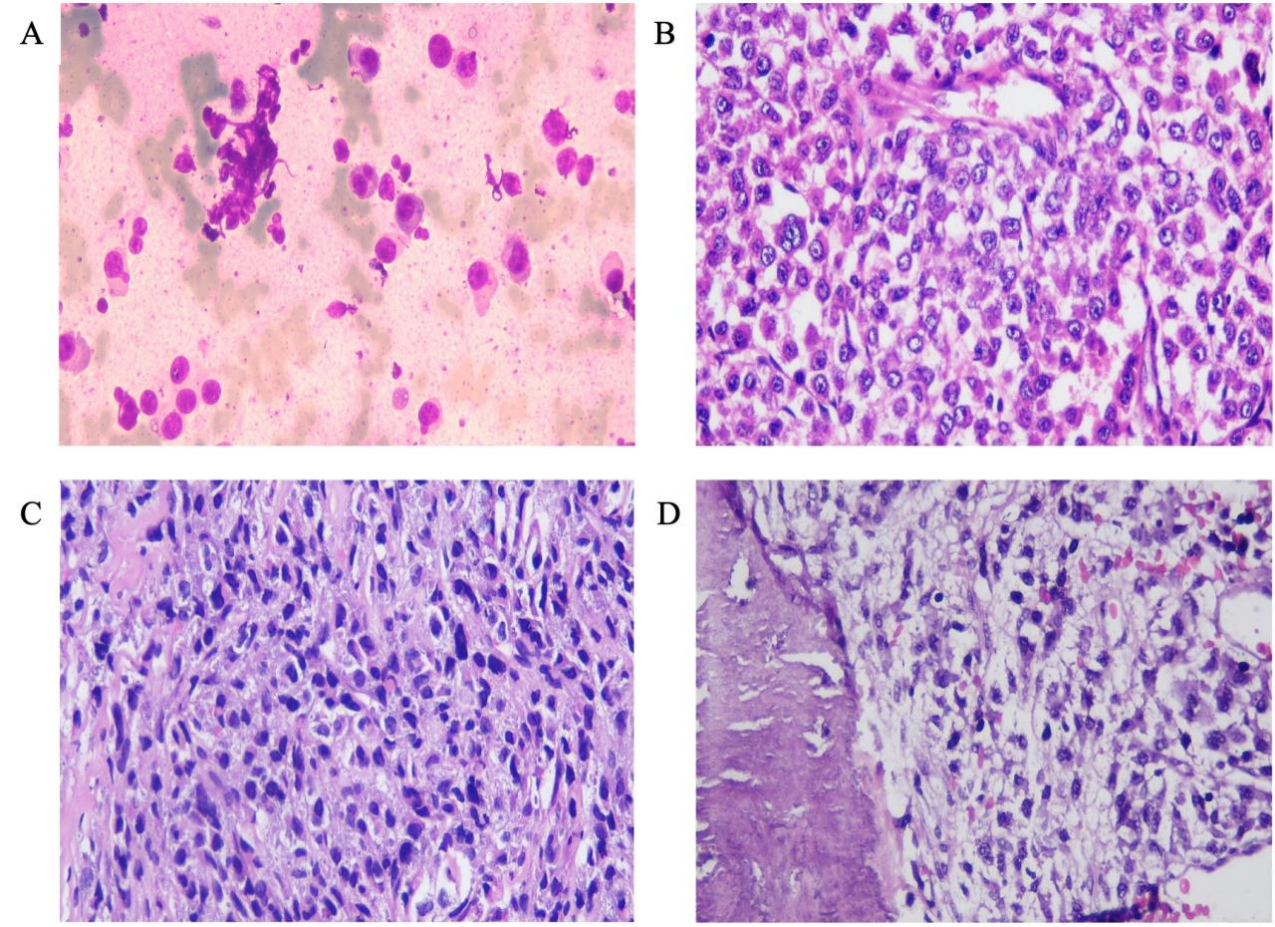
The smear slide of the FNAB examination shows (A) scattered atypical cells with eccentric nuclei, eosinophilic cytoplasm, and prominent nucleoli. (B) The cell block of FNAB shows a diffuse tumor pattern with similar cell characteristics. Hematoxylin-Eosin-stained section of left hemithorax soft tissue mass (C) and fracture sites of the humerus (D) show a diffuse arrangement of tumor cells, consisting of atypical plasmacytoid cells, infiltrating the surrounding area (magnification 40×).

Fine needle aspiration biopsy (FNAB) of all representative lesions showed atypical plasmacytoid cells (Figures 2A & 2B), suggesting MM. However, the serological laboratory profile, including serum calcium and albumin, urine Bence Jones protein level, creatinine level, and glomerular filtration rate,), were all within normal limits, which was inconsistent with the diagnosis of MM. The patient required surgical intervention to address multiple fractures and underwent a core biopsy procedure to investigate subcutaneous nodules. Histopathological examination of both subcutaneous nodules and bone samples revealed an infiltrative tumor of atypical epithelioid cells with eccentric nuclei. 

The clinical and radiological findings indicated the presence of a multiple-site tumor and eccentric nuclei cell tumor, suggesting a diagnosis of MM. However, it's important to note that other tumor types, like rhabdomyosarcoma, can also display eccentric nuclei, and some tumor cells may look like epithelioid cells. Therefore, other possible diagnoses, such as metastatic carcinoma and melanoma, were also considered (Figures 2C & 2D). Immunohistochemical analysis was requested for CD138 (a marker for MM), CK (a marker for metastatic carcinoma), vimentin, desmin, and myogenin (markers for rhabdomyosarcoma), and S100 and HMB45 (markers for melanoma). 

**Fig. 3:**
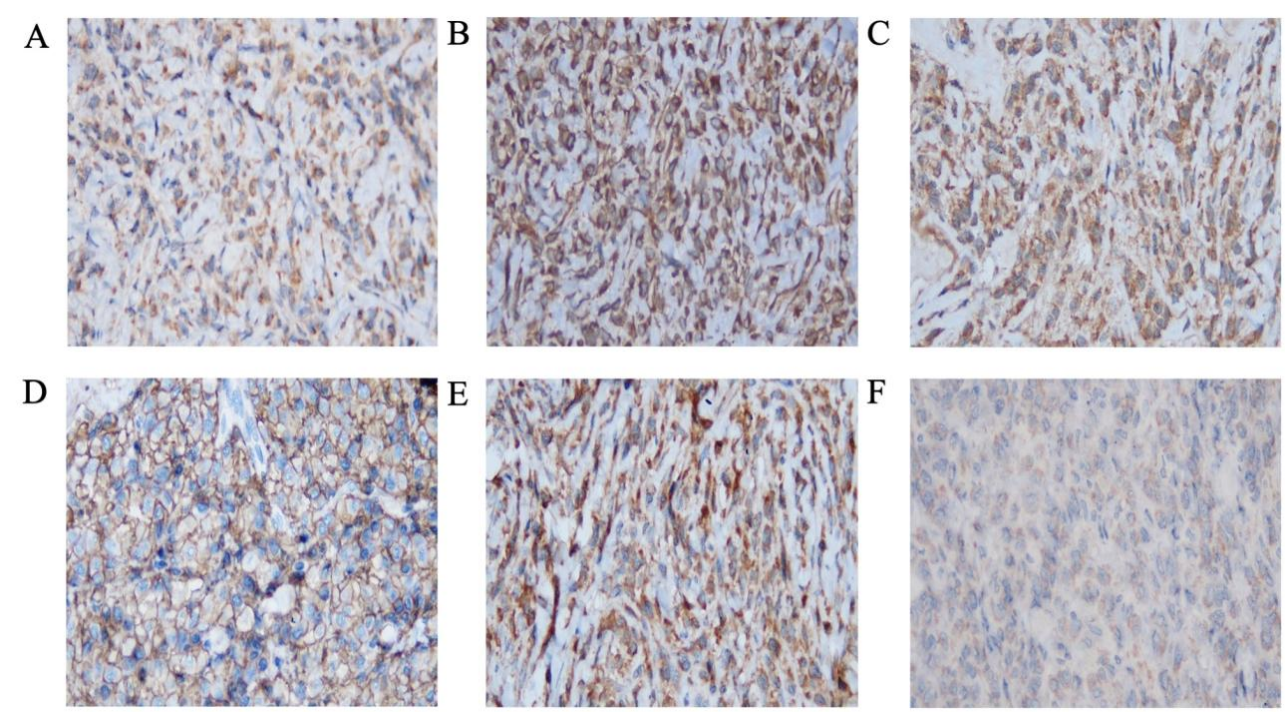
IHC results show positive staining for (A) CK, (B) vimentin, (C) Desmin (result not shown), and S100 in cytoplasm. (D) Positive staining for CD138 is seen in cell membranes. (E) Strong staining for HMB45 is seen in the cytoplasm. (F) Cell nuclei show negative staining for myogenin (magnification 40×).

Immunostaining results showed positivity for CK, vimentin, desmin, S-100, CD138, and HMB45. Myogenin staining was negative (Figure 3). During hospital admission, laboratory results showed a decreased calcium level (1.35 mmol/L) and normal tumor marker levels (AFP, CEA, PSA, and Ca 19-9). Routine evaluation showed an increase in calcium level (1.88 mmol/L), although it is still considered lower than the normal value. Other notable abnormal findings included low red blood cell count, hemoglobin, and hematocrit. Based on the clinical, laboratory, imaging, and pathology results, the diagnosis of amelanotic plasmacytoid melanoma was established. The laboratory and histopathology examinations were conducted during a hospital admission that lasted nearly a month. 

During hospitalization, the patient underwent open reduction with internal fixation for his fracture. Pre-surgical treatment includes intravenous ceftriaxone 1g/12 hours, ketorolac 30 mg/8 hours, and ranitidine 50 mg/12 hours. The patient was discharged after 2 weeks of hospital care in an improved condition with planned palliative chemotherapy in an outpatient setting. Other prescriptions were cefixime 1x200 mg, meloxicam 1x15 mg, and ranitidine 2x150 mg orally. However, the patient unfortunately passed away before any follow-up was able to be conducted.

## Discussion

Malignant melanomas may show a variety of cytomorphological characteristics, architectural patterns, and stromal alterations. Large pleomorphic cells, tiny cells, spindle cells, transparent cells, signet ring cells, pseudolipoblasts, rhabdoid cells, plasmacytoid cells, or even balloon cells may all be found in melanoma (6, 7). In a previous study in Brazil, 4 of the 12 patients with confirmed nasal melanoma showed mixed epithelioid and plasmacytoid features (8). Amelanotic melanoma, characterized by lacking pigmentation, also exhibits a wide range of clinical presentations and diverse histological appearances, earning it the reputation of being a master masquerade (9). Amelanotic melanoma with a plasmacytoid variation is a rare finding that can mimic many other conditions, particularly plasma cell proliferation (1, 3, 5). Owing to its rarity, plasma cell markers are expressed by plasmacytoid variants of melanoma, which may lead to a misdiagnosis of plasma cell proliferation. 

Previously reported melanoma cases with CD138 reactivity were old patients (age range, 50–78 years), consistent with the age of our patient (49 years). The locations of this entity were variable, including sinonasal, shoulder, elbow, bladder, and gastroesophageal junction. Our patient presented with multiple cutaneous nodules in the thoracic region and fractures (1-4, 9).

Immunohistochemical tests are crucial to provide an accurate and rapid diagnosis of malignant melanoma. Melanoma can express several aberrant immunophenotypic staining of another tumor type such as CK, desmin, glial fibrillary acidic protein, α-smooth muscle actin, carcinoembryonic antigen, epithelial membrane antigen, KP1, VS38, factor XIIIa, synaptophysin, and chromogranin (10). Some reports have indicated that CK protein expression in malignant melanoma is linked to unfavorable clinical outcomes (12). Furthermore, in a study, desmin expression was detected in 24% of melanoma cases, typically confined to a small number of cells (13). Additionally, almost all previous reports described CD138 expression in plasmacytoid melanoma (1-4). This explains the positivity of nearly all of the markers that had been checked. The absence of melanin makes the diagnosis more challenging and can lead to delayed diagnosis. The key to making the final diagnosis of melanoma is the immunostaining of melanocytic markers such as S-100, HMB45, and Melan-A (1, 10). The positivity of one melanocytic marker, such as S-100 and HMB45, can establish the diagnosis (13, 14). Even though immunohistochemical panels play an essential role in the diagnosis (2), the result must be carefully interpreted. The importance of the expression of CD138 in the biological behavior of melanomas is still obscure. Further research is required to obtain more insights (14). There is currently no scientific evidence indicating that the expression of CD138 impacts the response to treatment or the prognosis of amelanotic plasmacytoid melanoma.

The treatment approach for both melanotic (pigmented) and amelanotic (non-pigmented) melanoma is similar. Melanoma treatment options include surgery, radiotherapy, chemotherapy, and immunotherapy, each tailored to the stage and progression of the disease. Early detection and surgical excision of primary melanomas are critical for achieving favorable outcomes. When melanomas are identified at an early stage, surgical removal can often result in a cure. However, when the disease has advanced or spread to other parts of the body, chemotherapy is primarily used to manage the disease and provide palliative care, aiming to alleviate symptoms and improve quality of life. Research has highlighted a significant challenge with amelanotic melanomas, which are melanomas that lack the typical dark pigmentation. These colorless melanomas often go unnoticed and are diagnosed at more advanced stages, resulting in lower survival rates due to the delay in diagnosis and treatment (9, 10). 

A concerning complication in melanoma patients is the development of metastatic bone disease. Studies show that up to 17% of melanoma patients may experience this, where cancer cells spread to the bones, severely compromising skeletal integrity. This can lead to a range of skeletal-related events, such as painful fractures, spinal cord compression, and decreased mobility, all of which significantly diminish the patient's quality of life. These skeletal complications not only cause physical discomfort but also contribute to a decrease in survival rates. In our case, the patient developed metastatic bone disease, experiencing severe pain and mobility issues that greatly affected their daily life and overall prognosis, ultimately leading to a decline in health and survival (15). 

Approximately 40-60% of melanomas harbor a mutation in the BRAF gene, with the most common BRAF mutation in melanoma being the V600E mutation. Testing for BRAF mutations is crucial in melanoma patients to determine the appropriate treatment strategy for BRAF inhibitor administration. However, in this case, we did not perform BRAF mutation testing due to the limitations of our institution's laboratory facility. Personalized treatment approaches based on the genetic profile of the tumor allow for more effective and targeted therapies, improving survival rates and quality of life for patients (16).

It can be difficult to identify all melanomas, especially because some melanomas lack the typical brown or black pigmentation, obscuring the diagnosis. Immunostaining of S100 and HMB45 is very helpful in the diagnosis. Melanoma should be considered as a possible diagnosis in malignant subcutaneous soft tissue tumors with multiple bone fractures, as it also has plasmacytoid features with positive expression of CD138, mimicking MM.

## Funding/Support

The authors have no discord of interest to declare, and the Funincial support is University of Kufa.

## Data Availability

Data are available upon reasonable request from the corresponding author.
